# The Deep-Sea Natural Products, Biogenic Polyphosphate (Bio-PolyP) and Biogenic Silica (Bio-Silica), as Biomimetic Scaffolds for Bone Tissue Engineering: Fabrication of a Morphogenetically-Active Polymer

**DOI:** 10.3390/md11030718

**Published:** 2013-03-08

**Authors:** Xiaohong Wang, Heinz C. Schröder, Qingling Feng, Florian Draenert, Werner E. G. Müller

**Affiliations:** 1 ERC Advanced Investigator Grant Research Group at Institute for Physiological Chemistry, University Medical Center of the Johannes Gutenberg University, Duesbergweg 6, D-55128 Mainz, Germany; E-Mail: hschroed@uni-mainz.de; 2 National Research Center for Geoanalysis, Chinese Academy of Geological Sciences, 26 Baiwanzhuang Dajie, 100037 Beijing, China; 3 Department of Materials Science and Engineering, Tsinghua University, 100084 Beijing, China; E-Mail: biomater@mail.tsinghua.edu.cn; 4 Department and Clinic for Oral and Maxillofacial Surgery, Baldingerstraße, D-35033 Marburg, Germany; E-Mail: draenert@draenert.net

**Keywords:** scaffold, bone tissue engineering, bio-silica, bio-polyphosphate, morphogenetic scaffolds, BMP-2, osteoprotegerin, RANKL

## Abstract

Bone defects in human, caused by fractures/nonunions or trauma, gain increasing impact and have become a medical challenge in the present-day aging population. Frequently, those fractures require surgical intervention which ideally relies on autografts or suboptimally on allografts. Therefore, it is pressing and likewise challenging to develop bone substitution materials to heal bone defects. During the differentiation of osteoblasts from their mesenchymal progenitor/stem cells and of osteoclasts from their hemopoietic precursor cells, a lineage-specific release of growth factors and a trans-lineage homeostatic cross-talk via signaling molecules take place. Hence, the major hurdle is to fabricate a template that is functioning in a way mimicking the morphogenetic, inductive role(s) of the native extracellular matrix. In the last few years, two naturally occurring polymers that are produced by deep-sea sponges, the biogenic polyphosphate (bio-polyP) and biogenic silica (bio-silica) have also been identified as promoting morphogenetic on both osteoblasts and osteoclasts. These polymers elicit cytokines that affect bone mineralization (hydroxyapatite formation). In this manner, bio-silica and bio-polyP cause an increased release of BMP-2, the key mediator activating the anabolic arm of the hydroxyapatite forming cells, and of RANKL. In addition, bio-polyP inhibits the progression of the pre-osteoclasts to functionally active osteoclasts. Based on these findings, new bioinspired strategies for the fabrication of bone biomimetic templates have been developed applying 3D-printing techniques. Finally, a strategy is outlined by which these two morphogenetically active polymers might be used to develop a novel functionally active polymer.

## 1. Introduction

The inorganic, extracellularly located and assembled bone structures play crucial roles in human physiology, e.g., protection and support of soft tissue and organs, movement of the individual, mineral storage and in turn also blood pH regulation (see [1]). Furthermore, the bone elements act as a cavity for the different progenitor stem cells (mesenchymal and hemopoietic) and provide them with the required suitable environment (e.g., [2]). Therefore, it is very obvious that bone diseases and defects are crucially deleterious for the physiological and biochemical maintenance and homeostasis of the integrated organism. A series of bone manifestations/malfunctions, associated with the diseases of osteogenesis imperfecta, osteoarthritis, osteomyelitis, and—with increasing importance—osteoporosis, lead to bone defects that constitute tremendous clinical and economic significance. These bone diseases are frequently paralleled with traumatic injury as well as orthopedic surgeries and also tumor resection. The treatments of these pathophysiological bone symptoms/syndromes often involve bone repair or replacement. Those surgical interventions mostly cannot undergo self-repair via mechanical fixation alone and in turn do not allow the unification of the non-affected bone tissue surrounding the injured parts. As an example, statistics revealed that approximately 10% of the bone fractures connected with football injuries are not self-healing but require additional treatment [3]. As summarized [1], non-union processes in joint arthroplasties, primary tumor resection or massive traumatic bone loss, do not heal with mechanical fixation only. In those clinical signs, a stabile substitution material must be used to fill in the bone defect. In dependence on the size and the location of the defect, treatment with moldable implants or processed scaffold materials is required. Needless to mention that certainly autogenous bone grafting (coming from the patient’s own body) is the current “gold standard” for the treatment of critical-sized bone defects. Since those bone grafts are not sufficiently available, or in most cases cannot be taken from the patient, substitution materials have to be used instead. One alternative is allograft materials (coming from another individual of the same species). Those materials have lost most of their growth promoting and differentiation-inducing factors, moreover carry the risk of disease transmission or adverse host immune responses (reviewed in [4]). Finally also xenografts (coming from a non-human species) can be considered whose application is limited due to the perceived risk of disease or virus transmission and also infection as well as toxicity that is associated with sterilization, immunogenicity, and finally host rejection [5]. 

In principle, two different bone substitution materials can be used to replace the natural grafts; moldable bone substitution materials and scaffold-based bone materials. In order to contribute to a solution of this task, we used a “biomimetic” approach. We applied biological principles and systems used by nature, to study and to design novel engineering systems and novel intelligent materials (for a comprehensive review see [6]). The prime characteristics of such a biomimetic bone substitution material should be at least osteoconductive (able to guide cells, involved in the reparative growth of the natural bone, to the lesion) or better osteoinductive (stimulation of osteoprogenitor stem cells to differentiate into osteoblasts and triggering them to start with the formation of new bone), as described in [7]. In both cases, the healing process should result in osseointegration, a tight structural and functional connection and interaction between the existing living bone and the artificial implant. 

## 2. Two Novel Bio-Inorganic Polymers from Deep-Sea Sponges: Candidate Molecules for Biomimetic Bone Substitution Materials

Two bio-inorganic polymers, biogenic polyphosphate (bio-polyP) and biogenic silica (bio-silica), which have been considered as promising biomimetic bone substitution materials, are formed in plants, animals and some bacterial taxa and both gained interest in the recent few years [8,9]. These biologically formed polymers, identified both in deep-sea and shallow-water sponges, are in progress to take the hurdle from the molecular biological, biochemical, cell-biological level to preclinical studies. The state-of-the-art in progress of these polymers is given in this review. Both polymers are available in sufficient quantities, since both of the can be synthesized (bio)chemically. Bio-polyP is assumed to act as a co-polymer involved in bio-silica formation, while bio-silica represents the matrix for the formation of the skeletal elements of the siliceous sponges, for the spicules [8,9]. 

We focus on and introduce these two inorganic biopolymers, bio-silica and bio-polyP since they were recently proved to positively and favorably influence osteoblast growth and function and are considered to be applicable as new biomimetic bone substitution materials (reviewed in [8]). Both polymers are abundantly formed in nature, bio-silica in plants and animals (see [10]) and bio-polyP in both pro- and eukaryotic systems (reviewed in [11,12]). 

In most of our *in vitro* studies, we used two permanent cell lines, SaOS-2 cells and RAW 264.7 cells. SaOS-2 (sarcoma osteogenic cells of mesenchymal origin [13]) is a non-transformed cell line originating from primary osteosarcoma cells and retains a (limited) differentiation capacity [14,15]. RAW 264.7 cells is an osteoclast-like murine monocytic cell line [16,17]. These two cell lines have been successfully used to understand bone metabolism. 

### 2.1. Biogenic Polyphosphate (Bio-PolyP)

Inorganic polymeric phosphate, polyphosphate (polyP), can be chemically prepared either in a crystalline or an amorphous state [11,12]; the biogenic polyphosphate (bio-polyP) is amorphous [12,18]. Similar to silica/bio-silica, synthesis of polyP requires high temperature [19], while the biogenically formed bio-polyP is metabolically produced in bacteria and animals at ambient temperatures via kinases (see [12]). Bio-polyP is synthesized in a wide range of organisms, from bacteria, fungi and algae to plants and animals (see [12]). The polymer is readily soluble in water at millimolar concentrations with chain lengths below 100 phosphate units [20,21]. The natural bio-polyP is only found as a linear polymer, in which tens to hundreds of phosphate residues are linked by phosphoanhydride bonds. In the absence of any enzyme, the phosphoanhydride bonds within the polymer are stable over wide temperature and pH ranges [11,12]. PolyP had been and is successfully used as a food additive as well as a base material in the formulation of cosmetic products [22]. The nutritional benefit of polyP in animal food materials is well established [23]. Since bio-polyP exists as a multivalent anion, it binds to essential cations, such as Ca, Mg, Mn, Fe, and Co ions (see [12]). More specifically, bio-polyP acts as a strong Ca^2+^ chelator and also as an antioxidant [24]. 

The biological function of polyP has been studied to some details in microorganisms (reviewed in [25]) and more recently also in animals (reviewed in [12,26]). Based on experimental data, it has been proposed that bio-polyP acts as a storage substance of energy, as a chelator for metal cations, as an inducer of apoptosis, and—importantly—as a stimulating agent in mineralization of bone tissue [26,27,28] (reviewed in [11]). Likewise important, bio-polyP acts as a modulator of gene expression. Results [29] suggest that in the osteoblast-like cell line, MC 3T3-E1, bio-polyP causes an increased gene expression of osteocalcin, osterix, bone sialoprotein, and tissue non-specific alkaline phosphatase, all proteins known to be crucial for bone formation [30,31]. The gene expression data in MC 3T3-E1 cells have been obtained with 1 mM polyP [29,32]. 

Bio-polyP can increase skeletal mineralization process, but until today, it is still unknown whether this happens in its polymeric form or in monomeric phosphates which are formed from bio-polyP through hydrolysis by phosphatases [33]. The susceptibility of bio-polyP for enzymes, especially phosphatases is well established [34,35,36]. As one consequence of the enzymatic hydrolysis of polyP, a release of Ca^2+^ ions has been proposed; this cation is metabolically utilized during hydroxyapatite formation [18]. 

Very recently, it could be demonstrated that bio-polyP displays morphogenetic activity on bone-forming osteoblasts, SaOS-2 cells, and inhibitory activity on RAW 264.7 cells acting as osteoclasts. The osteoblast-like SaOS-2 cells form hydroxyapatite crystals, in response to exposure to bio-polyP, based on their potency to express key molecules known to control hydroxyapatite formation (see [37,38,39]), e.g., the bone morphogenetic protein 2 (BMP-2), an inducer of bone formation [40], osteoprotegerin (OPG), a cytokine that is expressed in osteoblasts with a significant role in the maturation of osteoclasts as well as in the control of bone mineral density [41], and the receptor activator of the NF-κB ligand (RANKL), a mediator that binds to RANK which is a receptor that mediates maturation of osteoclasts [42]. In turn, the relative concentration ratio between OPG and RANKL is crucial for the differentiation and survival of osteoclasts, since OPG can bind to RANKL and by that inactivates its function [7,38]. Hence, the OPG and RANKL ratio controls the osteoinductivity on the level of RANKL, a decisive ligand required for the differentiation of osteoclasts [43]. Likewise, the osteoclast-like RAW 264.7 cells have the potency to readily differentiate into osteoclasts when they are exposed to recombinant RANKL [44] and, by that, have been successfully used as a model for studies of osteoclastogenesis *in vitro* [45]. It is also important to mention that the activity of bio-polyP can induce alkaline phosphatase, an enzyme which provides inorganic phosphate required for the synthesis of hydroxyapatite [39]. 

In order to avoid any chelating activity of bio-polyP in our *in vitro* studies, we applied this polymer together with CaCl_2_ (bio-polyP•Ca^2+^ complex). This precaution excludes the possibility that the observed biological effects might be attributed to a depletion of Ca^2+^ ions that are required for hydroxyapatite deposition [46]. The bio-polyP•Ca^2+^ complex was found to be a strong inducer of hydroxyapatite formation in SaOS-2 cells and in particular, to cause an enhanced expression of *BMP-2* [47]. In parallel, bio-polyP•Ca^2+^ strongly inhibits the progression of RAW 264.7 cells into osteoclasts, which is reflected by the reduction of cells expressing tartrate-resistant acid phosphatase (TRAP), a well established marker protein for terminally differentiated osteoclasts [48]. As an additional endpoint marker for osteoclast differentiation, the effect of bio-polyP•Ca^2+^ on the function of IκBα kinase (nuclear factor of kappa light polypeptide gene enhancer in B-cells inhibitor, alpha) was determined. This kinase is one key molecule which causes the activation of NF-κB during RANKL-caused (pre)osteoclast differentiation (see [49]). The results revealed that bio-polyP•Ca^2+^ inhibited at low concentrations (10 to 100 μM) the phosphorylation, and by that, the signaling function of IκBα via the respective kinase in RAW 264.7 cells [47]. 

**Figure 1:**
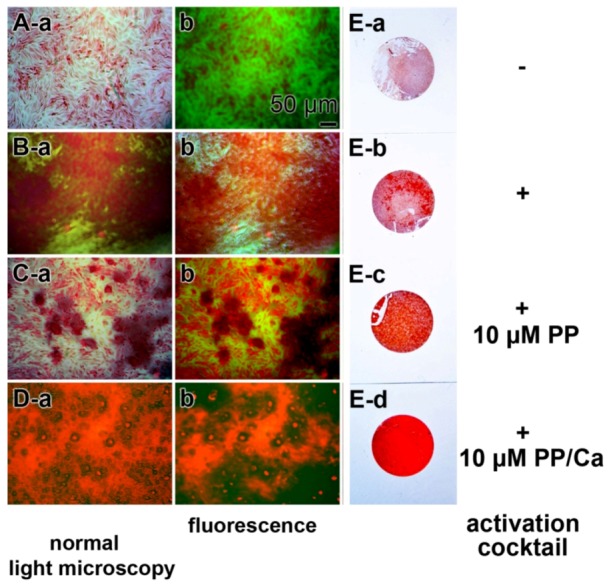
Stimulatory effect of bio-polyP, as a bio-polyP•Ca^2+^ complex on the process of mineralization of SaOS-2 cells *in vitro*. SaOS-2 cells were incubated in McCoy’s medium/FCS (fetal calf serum) for 2 days and, after attachment, transferred to McCoy’s medium/10% FCS and subsequently continued to grow for 7 days (**A**) in the absence (−activation cocktail) or (**B**–**D**) presence (+activation cocktail) of activation cocktail, composed of 5 mM β-glycerophosphate/50 mM ascorbic acid/10 nM dexamethasone, which is required for extensive hydroxyapatite formation. Where indicated, the assays were supplemented with either 10 μM bio-polyP (PP) or 10 μM polyP (Ca^2+^ complex) (PP/Ca). Then the specimens were stained with alizarin red S and the samples were analyzed by digital light or by fluorescence microscopy, as indicated. The red staining reflects the extent of hydroxyapatite mineralization. (**E**) In parallel, plastic coverslips were likewise stained with alizarin red S and documented/photographed as well.

The effect of bio-polyP on the biomineralization extent (hydroxyapatite formation) had been studied and the light microscopic images are given here (Figure 1). Incubation of SaOS-2 cells was performed for 10 days on plastic coverslips in 24-well plates, using McCoy’s medium/10% FCS (fetal calf serum) in the absence or presence of the activation cocktail, composed of β-glycerophosphate–ascorbic acid–dexamethasone [47]. The cultures which were grown in the presence of the activation cocktail were exposed to 10 μM polyP or 10 μM bio-polyP•Ca^2+^ for 10 days. Then the slips were stained with alizarin red S, a largely sensible indicator dye for hydroxyapatite visualization. An inspection of the cultures on the coverslips revealed, as expected, that the intensity of the staining was lowest in cultures grown in the absence of activation cocktail (Figure 1Ea). In contrast, cultures that were grown in the presence of activation cocktail showed a strong intensive staining, reflecting a higher level of hydroxyapatite mineralization (Figure 1Eb). If the cultures were treated with the activation cocktail and 10 μM polyP, no striking difference in the intensity of alizarin red S staining, compared with cultures grown with activation cocktail only, was observed (Figure 1Ec). However, if the cultures were grown in medium containing the activation cocktail and 10 μM bio-polyP•Ca^2+^, a strong and almost homogeneous staining of the cell layer was seen, a finding that reflects the strong hydroxyapatite formation by SaOS-2 cells (Figure 1Ed).

A closer inspection of the inducing effect of bio-polyP•Ca^2+^ by digital light microscopy revealed that the samples grown in McCoy’s medium/FCS showed only a scattered staining of the cell layer (Figure 1Aa). Likewise, the red staining was low if the samples were examined by red/green emitting fluorescent light (Figure 1Ab). A distinct increase in the red intensities was seen if the cells were cultivated in the presence of activation cocktail and then stained with alizarin red S in order to monitor the hydroxyapatite deposition (Figure 1B). The red patches (Figure 1Ba) as well as the red fluorescence areas (Figure 1Bb) strongly increased and extended over 50% of the visual fields. If the cells were incubated for 7 days in the presence of both the activation cocktail and 10 μM bio-polyP, the red patches/red fluorescence areas were smaller (Figure 1Ca,Cb) compared to the fields stained with alizarin red S in cultures grown with the activation cocktail only (Figure 1B). However, if the cultures were incubated with the activation cocktail together with 10 μM polyP (Ca^2+^ salt), an almost complete red staining was seen, reflecting an intensive hydroxyapatite mineralization (Figure 1D). These data show that the activation cocktail is required for extensive mineralization of SaOS-2 cells. Moreover, it is demonstrated that bio-polyP, if complexed with Ca^2+^ strongly augments the inducing effect of the activation cocktail on hydroxyapatite crystallite formation.

**Figure 2:**
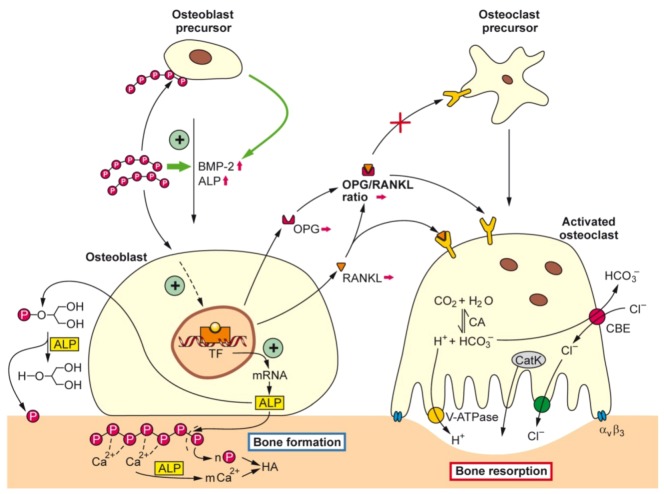
Schematic representation summarizing of the effect of bio-polyP, in form of the Ca^2+^ salt, on the tuned interaction between osteoblasts (hydroxyapatite anabolic pathway) and osteoclasts (hydroxyapatite catabolic pathway). It is outlined that bio-polyP supports the progression of precursor osteoblasts to mature osteoblasts by induction of the genes encoding BMP-2 (bone morphogenetic protein 2) and ALP (alkaline phosphatase), followed by the increased release of ALP. It is assumed that polyP activates a hypothetical TF (transcription factor). ALP is hydrolyzing both polyP (Ca^2+^ salt) and β-GP (β-glycerophosphate). The CBE (chloride-bicarbonate exchanger) in concert with the CA (carbonic anhydrase) is involved in the homeostasis of the intracellular CO2 concentration and pH level.

Taken these data together we show that bio-polyP affects the tuned balance between the osteoblasts and osteoclasts in the anabolic direction, implying that hydroxyapatite synthesis is favored at the expense of hydroxyapatite degradation/dissolution. A summary of the action modes of bio-polyP is given in the schematic presentation shown in Figure 2. 

### 2.2. Biogenic Silica (Bio-Silica)

The formation of the skeletal system from the earliest metazoans, the sponges (phylum Porifera) [50] to the crown taxa, the mammalians [51] and the insects [52], is dominated by a tuned communication between cells controlling anabolic processes and cells executing catabolic reactions. Basically, two kinds of inorganic scaffold materials had been applied in the metazoan kingdom to form skeletons: calcium (calcium-based skeletal systems) and—only found in siliceous sponges—silica (silica-based skeletons). 

A breakthrough in the understanding of the siliceous spicule formation of the demosponges and the hexactinellid sponges came from the discovery that the axial filaments of the spicules, the skeletal elements of these sponges (demosponges [53]; hexactinellids [54]) contain an enzymatically active protein which synthesizes polymeric silicate, the bio-silica. This enzyme, termed silicatein, has been found to catalyze/polycondensate bio-silica during the axial and radial growth of the spicules. In contrast to plant phytoliths and diatom frustules, where bio-silica is deposited from a super-saturated solution onto organic templates, the siliceous spicules of sponges are formed in a hypo-saturated intraorganism environment following an enzymatic mechanism by lowering the activation energy of the polycondensation reaction. In *in vitro* systems, orthosilicic acid or TEOS (tetraethyl orthosilicate) has been used as substrate/bio-silica precursor for the enzymatic reaction. During the latter process, ethanol is released [53]. The silicatein-mediated formation of silica proceeds at silica substrate concentrations of around 200 μM, far below the concentrations which are required for the chemical condensation of 1 mM or higher at neutral pH [55] (reviewed in [56,57]). 

The silicateins were first identified in the axial filament of the demosponge *Tethya aurantium* [58]. They comprise a family of related protein sequences which are consisting of three isoforms, silicatein-α, silicatein-β, and silicatein-γ, in a molar ratio of 12:6:1. The silicateins belong to the papain-like cysteine protease superfamily and are the most closely related to the cathepsin family [59]. The first cathepsin in sponges was identified and cloned from the demosponge *Geodia cydonium* [60].

## 3. Silica as an Essential Nutrient

**Figure 3:**
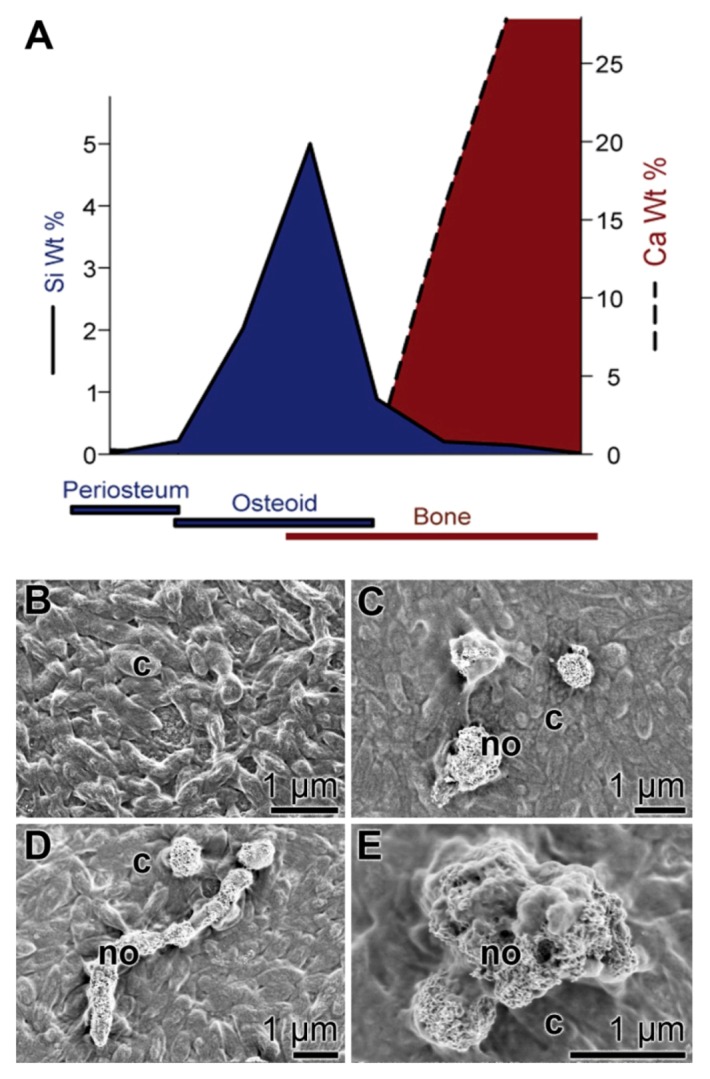
Biomedical approach for an application of bio-silica. (**A**) Schematic representation of the spatial relationship between silicon accumulation and calcium incorporation during early stages of bone formation in rats (modified according to Carlisle 1986). (**B**–**E**) Formation of hydroxyapatite nodules (bone hydroxyapatite depositions) on SaOS-2 cells; scanning electron microscopic images. The cells were grown in McCoy’s medium supplemented with FCS and the activation cocktail for inducing biomineralization. (**B**) Control cells (c) were cultivated on an untreated hydroxyapatite matrix. After 5 days in culture, no hydroxyapatite nodules are detected. (**C**–**E**) Cells (c) were grown onto a bio-silica coated matrix. After 5 days, distinct clusters of hydroxyapatite deposits, nodules (no), are formed.

Silica is an essential nutrient both for the natural ecosystem in general [61] and for humans and other vertebrates in particular [62,63]. Importantly, silicon deprivation results in severe skeletal malformations [64]. The experimental studies showed that in avian connective tissue, the highest silicon concentrations are found, in contrast to heart or muscle tissue, where the silicon concentrations are much lower. Moreover, a spatial correlation could be established between the areas of bone formation within animal tissue and the accumulation of silicon (Figure 3A). Based on these data, it had been concluded that a burst of silicon accumulation occurs around the osteoid and osteoid-bone interfaces, implying that this inorganic component is essential for bone formation. Consequently, we studied the effect of bio-silica on the activity of osteoblasts and osteoblasts *in vitro*. SaOS-2 cells were grown in the activation cocktail on a support, coated either with hydroxyapatite or with bio-silica. The cell layers that were grown on hydroxyapatite did not form hydroxyapatite crystals on their surfaces (Figure 3B), while the cells that had been cultivated for 5 days on bio-silica well formed hydroxyapatite crystals that are often fusing to clusters (Figure 3C–E; [38]). This observation which had been supported by alizarin red S staining assays underscores that bio-silica displays an inductive effect on SaOS-2 [37,38]. 

As outlined above, the tuned interaction of osteoblasts and osteoclasts during bone formation is well established. Furthermore, the decisive role of the osteoclastogenic ligand RANKL had been discussed also. This ligand is processed by metalloproteases to a soluble form and interacts with RANK and finally induces osteoclastogenesis (see [65]). This molecular cross-talk that coordinates osteoclastogenesis is controlled by the third component, OPG; this osteotropic effector acts as a soluble bone protector (see [66]). In turn, the molecular triad, OPG/RANK/RANKL (see [67]), is not only crucially controlling osteoclast differentiation, but is also involved in cell differentiation pathways of the immune and vascular systems (see [65]). *Vice versa*, recent results provided experimental evidence demonstrating that osteoclasts contribute with their cytokines to the fine-tuning of the osteoclast/osteoblast balanced functions (see [68,69]).

**Figure 4:**
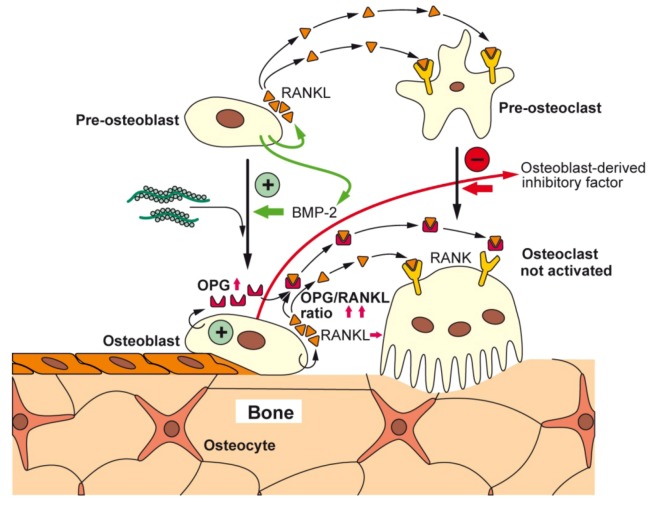
Proposed effects of bio-silica on osteoblasts, osteoclasts, and their progenitor cells; schematic representation. Bio-silica causes an increased expression of OPG in osteoblasts. In addition, the differentiation of osteoblasts is induced and accelerated by BMP-2. It is assumed that in turn the osteoblasts acquire the potential to differentiate to osteocytes and to lining cells. Furthermore, OPG counteracts various effects of RANKL, a cytokine that induces pre-osteoclast maturation and osteoclast activation. Finally, it could be identified that the osteoblasts release a factor, the osteoblasts-derived inhibitory factor that strongly inhibits the proliferation of osteoclasts; the nature of this factor is not yet known.

Following the established view that all metazoans originate from one ancestor, to which the siliceous sponges are the most closely related to [70], it had been postulated that the siliceous skeleton of sponges shares functional relationship to the Ca-based skeletons of vertebrates [50,71]. This view is supported by previous experimental evidence showing that silicate/silicon is an essential trace element in vertebrate nutrition [64,72,73]. In continuation, and sketched above we showed that bio-silica induces hydroxyapatite crystallite formation in SaOS-2 cells [74]. More recently, we demonstrated that in SaOS-2 cells, after exposure to bio-silica, a differential gene expression is seen that strongly up-regulates the steady-state level of *OPG* transcripts and leaves the level of *RANKL* transcripts almost unchanged [37]. Based on the observed increase of the OPG/RANKL ratio, it was intriguing to suggest that silica/silicate has a favorable biomedical potential for the treatment and/or prophylaxis of osteoporotic disorders [37]. Since silica was found to elicit *in vitro*, an increased (^3^H)dThd incorporation into DNA and a likewise increased HA formation, an osteogenic potential of silica had been deduced [38]. Finally we discovered that SaOS-2 cells, after exposure to bio-silica, release a soluble factor, the “osteoblasts-derived inhibitory factor”, which causes an inhibition of RAW 264.7 cell growth [75]. A schematic outline of the stimulatory effect of bio-silica on osteoblasts and the inhibitory action on osteoclast is given in Figure 4.

## 4. Moldable Implants

In a recent review, the characteristics of a moldable material, activating on bone-forming cells and using bio-silica as one component, have been summarized [76]. Based on our finding that the natural product, bio-silica, comprises osteoinductive activity, we formulated a two component moldable material. Silicatein was encapsulated together with its substrate, ortho-silicate, in poly(D,L-lactide)/poly(vinyl pyrrolidone)-based microspheres, termed silicatein-and-silica-containing microspheres (SSMs). Subsequently, the SSMs were successfully embedded in a poly(vinyl pyrrolidone)/starch-based matrix, termed plastic-like filler matrix containing silicic acid (PMSA). A blend of SSM and PMSA forms a biocompatible, moldable, and biodegradable functional implant material that hardens at a controlled and clinically suitable rate within approximately 30 min to 6 h to implants that were tightly integrated in artificial defects of rabbit femurs. Until now, no toxic reactions caused by the silicatein have been observed *in vitro* or *in vivo*, findings that led us to propose that the silicatein/bio-silica-based implant materials might have a beneficial potential in the field of regenerative medicine.

## 5. Scaffold

As outlined above, autogenous bone grafting is the hitherto not reached the highest reference quality standard for bone repair interventions of critical-sized defects in bone in spite of the intensive efforts in the field of bone tissue engineering. Synthetic bone scaffolds have—theoretically—significant advantages over allogenous bone grafts because they are not fraught with uncertainties, e.g., disease transmission, or risk of infection or immunogenicity. The prerequisites of synthetic bone scaffolds, to be effectively used for biomedical repair of bone defects, are (1) to mimic the physiochemical characteristics of the bone, a complicated challenge, and (2) to be associated with or to be endued with the properties to actively attract the bone constructing cells, either the progenitor cells or the functionally active terminally differentiated bone-forming cells (reviewed in [1,6]). 

### 5.1. Organic Scaffold: Osteoconductive/Osteoinductive Properties

Due to their excellent tissue compatibility and property, the physiologically bio-degradable substitution materials from natural origin, e.g., collagen [77], or silk fibroin [78], or derived and processed natural biomaterials/biopolymers, e.g., chitosan [79], starch [80], or poly(3-hydroxybutyrate) [81], and finally also totally synthetic polymers such as poly(lactide) [82], poly(lactide/glycolide) [83], and polycaprolactone [84], have been developed. These biopolymers mimic the supra-molecular structures of the natural extracellular matrix of tissues and have the very suitable biocompatibility characteristics of human bone. Also their porosity and fibrous network allow a “homing” for both the progenitor and the differentiated bone cells. 

The substitution materials based on demineralized bone matrix derived from human tissue provide a suitable advancement of the grafting material for repairing bone defect, since they retain the ability to act not only osteoconductively, allowing the circulating bone cells to attach and to proliferate but also to be osteoinductive [85], because they still contain bone growth factors, e.g., BMP-2, TGF-β (transforming growth factor beta), IGF-II (insulin-like growth factor 2), or PDGF (platelet-derived growth factor), even though in different concentrations [86]. Recently, a collagen microencapsulation technology had been described, by which bone marrow-derived mesenchymal stem cells had been entrapped into a biomimetically fabricated collagen fiber meshwork. This injectable material (microspheres) had been studied *in vitro* and found to display not only osteoconductive but also osteoinductive properties [87].

Focusing on processed, natural biomaterials, e.g., chitosan, those biopolymers are naturally lacking of these bone growth factors which have to be added after fabrication [88]. Likewise biologically or morphogenetically inactive materials are the totally synthetic polymers. This fact is not plausible since those polymers are lacking of the ligands required for the interaction with the receptors on the bone cell surfaces in order to induce the intracellular signaling cascade(s). Those stimulations by specific transcription factors are the clue for any cell- and tissue-specific differentiation process resulting in a functional and spatial interaction of cells to produce the extracellular bone structure. 

### 5.2. Inorganic Scaffold: Space-Filling Properties

Pure chemically prepared and fabricated bone materials, e.g., calcium phosphate, calcium sulfate or coralline carbonate and phosphate grafts, are suitable as osteoconductive implants, since they provide stability to the damaged bone. Hence they act osteoconductively and to some extent also osteointegratively, but they are lacking of any osteoinductive properties (see [89]). To take advantage of their excellent mechanical properties, these materials have to be biologically functionalized (reviewed in [6]). 

A similar functionalization with biological ligands has to be performed with titanium and its alloys that are widely used as orthopedic and dental implant materials. The major challenge is the mismatch which exists between the mechanical properties of the implant material with the bone tissue [90]. One established solution is the development of adjusted Ti/Nb/Zr/Sn titanium alloys whose Young’s modulus is close to the physiological Young’s modulus of approximately 50 GPa [91]. Two attractive functionalization procedures have been described, first the immobilization of the surface with RGD-ligands [92] and second, tailoring/patterning the surface of the titanium material with nano-spikes which fit in the architectural arrangement of the integrin receptors of the bone cells by which a functional morphology and an expression of growth factors in bone cells are elicited [93]. 

### 5.3. Bio-Inorganic Scaffold: Osteoinductive Properties of Bio-PolyP and Bio-Silica

**Figure 5:**
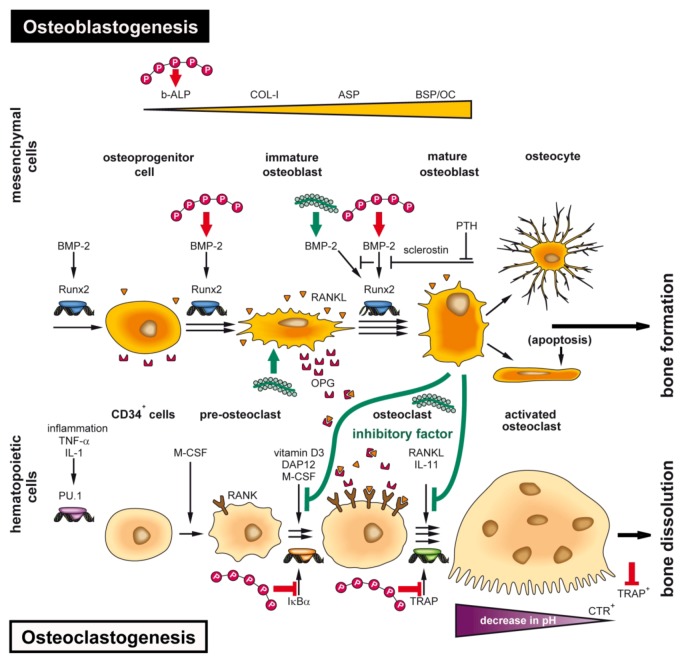
Schematic outline of the differentiation steps of the precursor/stem cells of the osteoblasts (osteoblastogenesis) and osteoclasts (osteoclastogenesis) with the main focus on the factors that cause an increase in hydroxyapatite formation. Upper panel: Osteoblast differentiation starts from the mesenchymal stem cells. This lineage ends with the osteocytes. The major transcription factor Runx2, which is under the control of BMP-2, is synthesized in chondrocytes and causes a stage-dependent increase in the structural and functional proteins, for example, b-ALP, COLI, OP, ASP and also RANKL, BSP and OC, in osteoblasts. The osteocytes become either finally embedded in the HA deposits and release sclerostin, a PTH-inhibitable glycoprotein which inhibits BMP-2, or the osteoblasts undergo apoptosis. Lower panel: Principle differentiation stages from the hematopoietic stem cells, via pre-osteoclasts to functionally active, bone-resorbing osteoclasts. The osteoblasts direct the pre-osteoclasts to the osteoclasts through RANK/RANKL, an interaction that is blocked by OPG. The osteoclasts start to differentiate from hematopoietic stem cells (circulating mononuclear cells) through activation of the PU.1 transcription factor and inflammatory signals. The CD34 osteoclast precursor cells, after entering the circulating system and the presence of macrophage-colony stimulating factor (M-CSF) and 1,25-dihydroxy-vitamin D3, become recruited onto the surface of bone. The pre-osteoclasts, after the stimulation of the DAP12 adapter protein/receptor undergo multi-nucleation to the osteoclasts. Those cells express in the presence of dihydroxy-vitamin D3, the receptor RANK. After binding of RANKL to RANK, the osteoclasts dissolve hydroxyapatite by lowering the pH. Markers for the activated osteoclasts are TRAP, CTR, and integrin a_v_b_3_. The inducers/activators of the differentiation steps are outlined in the text. The sites at which bio-polyP and bio-silica interfere (activate or inhibit) the differentiation pathways are highlighted in red (bio-polyP) and in green (bio-silica). The factor that is released by mature osteoblasts in the presence of bio-silica and inhibits growth/differentiation of pre-osteoclasts illustrates the cross-talk between osteoblasts and osteoclasts.

The functional interaction of osteoblasts with osteoclasts can be impressively described with reference to the widespread disease, osteoporosis; this degenerative bone disease causes loss of bone tissue and reduction of bone density which are reflected by a micro-architectural deterioration of the bone [94,95]. The cellular basis for this disorder is an imbalance between the bone-forming osteoblasts and the bone-resorbing osteoclasts. Even though the bone forming and bone resorbing cells differentiate from different cell lineages, their functions *in vivo* are intimately linked and their differentiation levels are reciprocally controlled [96]. The major transcription factor involved in the differentiation and proliferation of osteoprogenitor cells is Runx2, a factor that is expressed in the mesenchymal stem cells and along the different stages of the osteoblast lineage [97]. Runx2 itself is under the control of BMP-2 [98]. These inducer factors cause a stage-correlated and increasing expression of a series of genes, for example, of those encoding the bone-specific alkaline phosphatase (b-ALP), collagen type I (COL-I), osteopontin (OP) and—at a later stage—of RANKL, asialoprotein (ASP), bone sialoprotein (BSP) and osteocalcin (OC). The osteoblasts that produce hydroxyapatite, finally differentiate to osteocytes that remain entrapped in the hydroxyapatite deposits or undergo apoptosis [99]. In a feed-back loop, the osteocytes express sclerostin which functions as a potent antagonist of BMP-2. This effect can be counteracted by the parathyroid hormone [100] (Figure 5).

In contrast to osteoblasts, osteoclasts are multi-nucleated cells that originate from the hematopoietic lineage [101,102]. Those stem cells undergo differentiation and maturation in the presence of the macrophage colony-stimulating factor (M-CSF) and of RANKL. As markers for the multi-nucleated osteoclasts, the high expression levels of TRAP, of the calcitonin receptor (CTR) binding protein as well as of the expression of integrin a_v_b_3_ have been used [103] (Figure 2 and Figure 5). The above mentioned cytokine/receptor triad crucially controls bone formation and bone remodeling, RANKL with its receptor RANK and the endogenous decoy receptor OPG [104,105]. RANKL is synthesized by the osteoblastic lineage cells and is essential for the differentiation of those cells which are involved in bone resorption, the osteoclasts. RANKL is expressed on osteoblasts, T cells, dendritic cells, and their precursors, from where it can be released by specific proteases [106]. After binding of RANKL to RANK, the osteoclasts become activated and resorb bone mineral. During this process, the cells have close contact to the bone surface [107]. At this interphase, the bone vesicles are formed, via integrin (a_v_b_3_) which contain proton pumps and acid hydrolases (cathepsin K) (Figure 2). Those enzymes and vesicles are inserted into the cells at the bone-apposed area under formation of a “ruffled border”. A “resorptive hemivacuole” is formed between cell and bone, allowing the protons to dissolve hydroxyapatite of the bone (Figure 2). The intracellular pH is kept at a near-neutral level by chloride/bicarbonate exchange and the help of carbonic anhydrase [108].

To highlight again, the activity and function of RANKL is under control of OPG, which is secreted by stroma cells and also by osteoblasts [109]. OPG scavenges RANKL by binding to it and neutralizes its function. From these results, it is pressing to conclude that any deregulation of the tuned expression of the RANKL/RANK/OPG system causes a dysregulation of the differentiation pathways of the osteoblasts and the osteoclasts and in turn impairs bone remodeling [104]. More specific, OPG abolishes the activation of the osteoclast via inhibition of the RANK pathway and by that prevents bone matrix from excessive resorption. Hence, the relative concentration of OPG and RANKL in bone is the major morphogenetic determinant of bone mass and strength. 

*In* *vitro* and *in vivo* studies have been performed to counteract the age-correlated OPG-RANKL imbalance [110]. It has been found that the OPG-RANKL ratio is influenced by a series of substances, including hormones and cytokines. In the course of these studies, it could be established that treatment of osteoblasts with stimulators of osteoblast formation (e.g., vitamin D, PTH, prostaglandin E2 or interleukin-11) upregulates the expression of RANKL. Addition of BMP-2, interleukin-1b or TNF-α to those cells increases the OPG mRNA steady-state level and subsequently the OPG protein synthesis in fetal osteoblasts. Exactly here, at this regulatory step, the two biopolymers interfere with the relative RANKL/RANK/OPG levels. In response to bio-silica, the relative expression level of OPG increases [37] and as deduced from the existing data in the literature reduces the functional activity of RANKL.

The biopolymer bio-polyP inhibits the phosphorylation of the IκBα kinase in RAW 264.7 cells, and by that prevents the progression/differentiation of the pre-osteoclasts to the mature osteoclasts [47]. In addition, bio-polyP inhibits the expression of TRAP, amplifying the effect of this polymer on the kinase [47]. Finally it should be mentioned that a new inhibitory cross-talk effect is caused by bio-silica, which is centered in a hitherto not yet characterized inhibitory factor. 

These modes of inhibiting/activating interventions of the two biopolymers, bio-polyP and bio-silica, are sketched in Figure 5. 

### 5.4. Bio-Inorganic Scaffold: Formation of a Flexible Structure

It is the aim of a fabricated scaffold to provide the surrounding cells a three-dimensional (3D) platform onto which bone cells can grow, differentiate and finally deposit hydroxyapatite. Ideally the scaffold is composed of natural fibers which build structures to meet this requirement; those compositions are found in the extracellular matrix (ECM). The dominant structural protein in the ECM is collagen [111]. It builds the 3D structure with nanofibers which have a diameter between 50 and 500 nm [112], and forms the founding architecture for the growing bone. It is collagen type I that forms the structural, organic building of the bone to 90% [113]. There, collagen acts as the nucleation site for the growth of the highly ordered crystals, formed of carbonated apatite [Ca_5_(PO_4_,CO_3_)_3_(OH)]. Initially they are only a few nm thick [39]. As the skeletal structure, acting in nature as support for soft tissue [71], bones represent a hybrid material which makes them mechanically flexible, strong, stiff, tough and also lightweight [114]. 

The fabricated scaffold designed to mimic the natural inorganic/organic 3D bone structure must follow the nanofibrous architecture and must be engineered with high porosity in order to allow an ingrowth of cells and—in addition—an efficient transport of morphogens, cytokines, growth factors and also nutrients, oxygen as well as waste products (see [115]). A further characteristics and prerequisite for an efficient and functional scaffold is the vascularization of the inserted materials in order to avoid necrotic processes in the center of larger constructs. In turn the materials to be used as a biomimetic scaffold, either formed of an organic ground substance or of metal (titanium), must meet the acting mechanical stresses that occur during tissue neogenesis. Besides of being mechanically suitable, the materials must be osteoconductive, e.g., by using ceramics [116]—or ideally even osteoinductive. Furthermore, the material must be (minimally) biodegradable in order to circumvent the development of immune response and to allow new physiological bone cells to invade, grow and differentiate followed by the formation of an organic fiber network, and finally the full bone tissue regeneration. 

Besides of using nanofibrous scaffolds, prepared by molecular self-assembly, bacteria derived hydrogels, or by thermally-induced phase separation (reviewed in [115]), bone substitution materials can be prepared (1) by electrospinning or (2) by three-dimension (3D) printing. The electrospinning process allows the fabrication of 3D materials by using nanofibrous matrices that comprise some structural similarities to the physiological ECM at low cost (reviewed in [117,118,119,120,121]). This process takes advantage of electrostatic forces to generate polymer fibers. Typically the fibers have diameters of 100 to 500 nm to form nanofibers. Often the synthetic organic material contains PLA (polyglycolic acid) and PEG [poly(ethylene glycol)] also together with natural organic polymer e.g., chitin derivatives [121]. 

3D printing is a—relatively straightforward—technique by which a binding solution is printed into layers of powder, a process that is computer-controlled and imitates a sliced virtual model [122,123]. This technique had been successfully used to develop bone substitution materials with optimized integration and functionality characteristics [124]. Those implants can be tailored to a given individual defect 3D geometry, following the anatomical data information obtained from the patient. Especially calcium phosphate and bioactive glasses have been used as suitable starting materials for the fabrication of the 3D structures [125]. Usually the fabrication of 3D printed bone substitution materials involves a sintering step after an initial aggregate formation of the calcium phosphate and bioactive glass granules. An improvement, with respect to the (partial) elimination of the higher temperature step, was the development of the advanced microdispensor Ultimus technology, by which both fibrin scaffold and PLA meshes were prepared [126]. Likewise, also the layer-by-layer printing of 3D tri-calcium phosphate required a post sintering phase [127]. Despite this disadvantage, those scaffolds are promising materials allowing the osteoclastic cells to differentiate properly; in addition, this material is resorbable. 

In order to fabricate 3D scaffolds containing bio-organic polymers, e.g., collagen as supporting fiber, and avoiding any denaturating temperatures, the solid freeform fabrication (SFF) technique [128] coupled to the indirect 3D printing technique [129] was developed. In an exemplary procedure, a collagen scaffold was prepared by using the coupled SFF/indirect 3D printing technique [129]. Tendon collagen type I was dispersed in acetic acid buffer (pH about 3). Using this material, the scaffold was prepared following the SFF strategy [129,130,131]. At first, a computer-simulated negative mold was designed into which the collagenous solution was poured, followed by freezing at −30 °C. Finally, a dehydration and in turn a dissolution process was followed and by that a negative mold with a predefined microchannel network was created. After a critical point drying and cross-linking, the scaffolds was obtained. 

### 5.5. Bio-Inorganic Bio-Silica Scaffold

The inorganic bio-silica scaffold of siliceous sponge spicules is distinguished by its origin, enzymatically formed compared to all other bio-silica deposits. This origin implies that the nanoparticles formed by silicatein are spatially arranged and deposited via an enzyme-guided pathway [132]. Even more, the enzyme itself, silicatein is not only a catalytically active protein, but also a structure-giving backbone for the primordial/growing bio-silica fibrils [133]. Studying the maturation process of silicatein on a molecular level if it became overt that the enzyme acquires these properties (to be enzymatically active and to be structure-guiding) during the maturation, from the pro-silicatein to the active enzyme, a process during which the propeptide is split off from the inactive precursor [134]. During this hydrolytic cleavage, the active enzyme undergoes a conformational change that the molecule becomes less soluble and precipitates. This effect, which is seen at higher temperature (around 20 °C) results in the formation of self-assembled structures of silicatein, passes a fractal stage. The precipitated silicatein molecules can be re-dissolved in the presence of urea, without losing the biological activity. Those self-assembly formations are stabilized by addition of silintaphin-1 [135], a natural interactor of silicatein, and of PEG, a synthetic polymer. Adding the latter two components (silintaphin-1 and PEG) to the silicatein-mediated enzymic reaction results in the formation of biosilica cubes; those deposits become flat/planar and hard. The bio-silica product increases its compactness if silicatein is supplemented with silintaphin-1 or PEG. The elastic modulus of the silicatein-mediated biosilica product increases in parallel with the addition of silintaphin-1 and/or PEG from 17 MPa (silicatein) via 61 MPa (silicatein:silintaphin-1) to 101 MPa (silicatein:silintaphin-1 and PEG). This increase in hardness, in the presence of PEG, makes the structure-guiding enzyme, silicatein, suitable for the fabrication of controlled, pattern-forming structures. 

**Figure 6:**
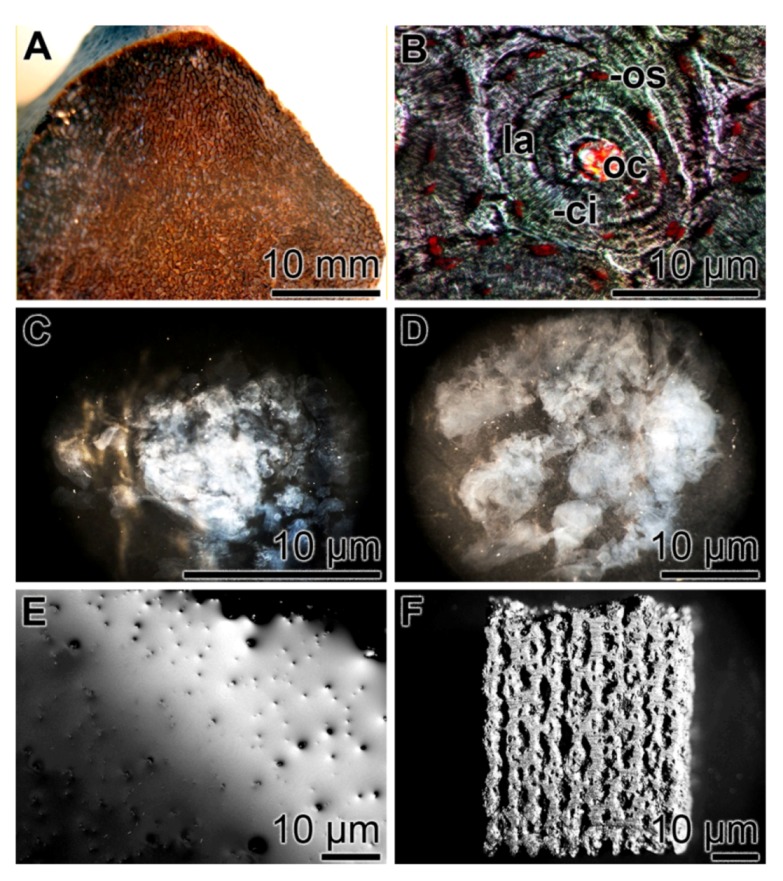
Fabrication of a 3D printed bio-silica scaffold from the enzymatically formed and loosely composed bio-silica product. (**A**) Vertebrate femur [*Rhamphorhynchus* sp. dinosaur (thigh bone), 150–148 Ma; Late Jurassic]; (**B**) Vertebrate bone osteon, composed of about 10 concentrically arranged lamellae (la) around a central opening, the osteonal canal (oc). In addition, the canaliculi (ci) associated with the osteocytes (os) are seen. (C to E) Hardening of the bio-silica product, enzymatically formed *in vitro* using the sponge silicatein. In a time-course experiment, the enzymatic bio-silica product had been supplemented with PEG (molar ratio to bio-silica of 1:0.1) and inspected with an optical microscope after 10 min (**C**), 30 min (**D**) and 60 min (**E**). During this process, the bio-silica material became flat and stiff. (**F**) A final bio-silica material formed via the computer-aided 3D printing technique.

We are applying these inherent properties of silicatein for 3D bio-silica/silicatein scaffolds fabrication via an indirect printing approach, a SFF/indirect 3D printing technique. The mold for the bio-silica scaffold was designed using computer in order to fabricate the negative mold; bone specimen from a vertebrate femur was used as a template (Figure 6A). In the initial experiments, we did not apply this new technique for the detailed osteon structures (Figure 6B) in order first to establish the proof of principle. In this new process, bio-silica was synthesized first in the presence of silicatein [133]. After the 1 h incubation period, the enzymatic reaction was terminated and the bio-silica product formed was supplemented with PEG and bio-silica at a molar ratio of 1:0.1. The bio-silica/PEG reaction mixture was inspected after 10 min (Figure 6C), 30 min (Figure 6D) and 60 min (Figure 6E) with an optical microscope. It is apparent that the initially formed fluffy bio-silica material increases in stiffness (Figure 6C,D) and the bio-silica polymer formed became finally homogenous and plane-structured (Figure 6E). This process—the hardening of the loosely formed initial bio-silica to the structured PEG/bio-silica—was applied to form the 3D printed bio-silica scaffold (Figure 6F). 

Parallel to this SFF/indirect 3D printing technique, we have also successfully applied the direct 3D printing technology. We followed the strategy that had been described for starch [136]. In brief, the starch scaffold was prepared and hardened. Then the 3D fabricated starch scaffold was incubated both with silicatein and its substrate ortho-silicate. Finally the framework was hardened by spraying with ortho-silicate together with PEG at a molar ratio of 1:0.1. For printing, the apparatus ZPrinter 450 (ZCorporation, Rock Hill, SC 29730; USA) had been used. The compressive strength and the stiffness of the sample were tested, and values of approximately 27.4 MPa (average compressive strength) were determined.

### 5.6. Biocompatibility of the Bio-Silica Scaffold

The biocompatibility of the bio-silica is excellent and no toxicity was determined *in vitro*, applying the MTT-viability assay. In addition, the requirement with respect to the porosity allowing a suitable assembly and growth of bone cells within the cavities of the scaffold was analyzed. The histological examination revealed that the cells within the scaffold showed proliferation and differentiation and—after addition of the mineralization cocktail—formed hydroxyapatite crystals. Furthermore, the scaffolds displayed a degradation at the physiochemical-biomimetic environment, in the cell culture medium/serum after 12 days. These analyses supported the view that the porosity of the material is suitable for the infiltration of cells and for a physiological interaction of the cells within the cavities. The pore sizes are within the range of 80–140 μm. 

### 5.7. Bio-Inorganic Bio-PolyP Scaffold

In initial studies, we could demonstrate that the biogenically and morphogenetically active bio-polyP is a likewise ideal polymer to be used for building of scaffold materials. Especially the chemical properties to be soluble at physiological pH conditions as a salt with the cations Na^+^ and K^+^, while becoming insoluble with cation Ca^2+^, provides this material an essentially suitable property to undergo hardening after the 3D printing process. Furthermore, the Ca^2+^ salt of polyP is—like the Na^+^ and K^+^ salts—biologically active and causes an increased release of the cytokine BMP-2. 

## 6. Future Direction: Application of Bio-Silica and Bio-PolyP in Bone Tissue Engineering

As outlined before [1], the advantages to utilize synthetic bone scaffolds include: the elimination of disease transmission risk, fewer surgical procedures, a reduced risk of infection or immunogenicity, and especially the abundant availability of synthetic scaffold materials. The basic challenge to develop a suitable synthetic scaffold is to mimic the complex physiological environment in which bone cells grow and differentiate. In a physiological framework, the bone cells find a suitable scaffold that allows them to ingrow into a scaffold with the matching porosity where they can differentiate and communicate sby signaling with the neighboring cells. Moreover, these cavities must allow the substrates for the osteoblasts to enter and to be available for the osteoid deposition, allow vascularization, and finally bone in-growth. 

**Figure 7:**
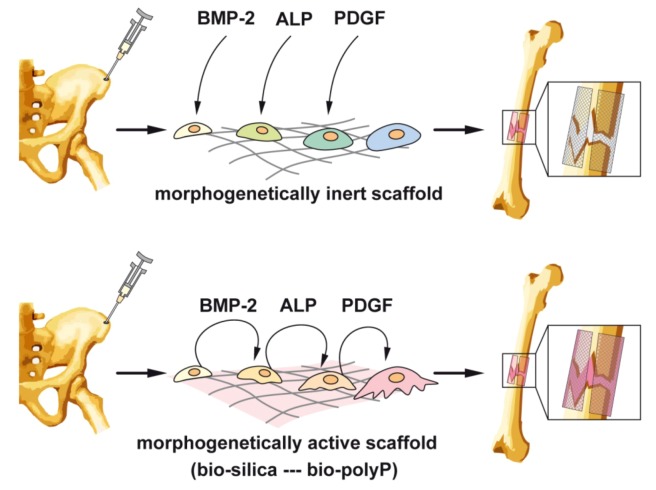
Outline of a strategy to prepare cell-populated scaffolds from cells, taken from e.g., iliac crest and cultivated *ex vivo* on either morphogenetically inert scaffold (upper panel) or on morphogenetically active scaffold, prepared from bio-silica or bio-polyP (lower panel). While in cultures with the inert template, the cytokines/factors [BMP-2 (bone morphogenetic protein-2), ALP (alkaline phosphatase) or PDGF (platelet-derived growth factor)] have been added from external sources, at least BMP-2 and ALP are actively elicited from the cells, resulting in a directed differentiation to functionally active osteoblasts.

Focusing on bone tissue engineering strategies, e.g., such as cell transplantation, a-cellular scaffolds, stem cell therapy, again the physiological regulatory network of cytokines and growth factors must be provided to the mesenchymal stems cells (MSCs) after the removal from the donor *ex vivo*. As sketched in Figure 7, the MSCs are taken from the donor, often from the iliac crest, and seeded onto a scaffold, where they must be cultivated and expanded. Here a major hurdle must be jumped in a way that the cells must be stimulated in the growth medium with the factors triggering the pluripotent MSC into the differentiation direction towards osteoblasts, provided with the ability to deposit hydroxyapatite. For this process, surely the mineralization cocktail, dexamethasone, ascorbic acid, and β-glycerophosphate must be added as the terminal “mineralization factors/substrates”. The addition of these substances is straightforward, and their price cheap. However, the cultures must be supplemented with the morphogens which had to be added at the phase-specific and appropriate differentiation stage. Among those are the relevant growth factors PDGFs, BMPs, IGFs, and TGF-βs. It would be ideal if the bone cells themselves, growing onto the artificial scaffold(s), are producing these factors timely and spatially in a correct pattern to allow a functional differentiation of the bone cells. As outlined in Figure 7 (upper panel), those factors have to be added to the cultures during the *ex vivo* expansion from external sources. This means that the scaffold is morphogenetically inert. 

The available data gathered in the last years indicate that the natural inorganic polymers bio-silica and bio-polyP, both abundantly produced in deep-sea sponges, display inductive activity and elicit from the bone cells the morphogens/ligand molecules BMP-2 and RANKL and by that cause a differentiation of the bone cells towards an anabolic, hydroxyapatite-forming status (Figure 7; lower panel). Certainly these data are only the first step towards the development of a morphogenetically-active polymer suitable to function alone as template/modulator for cells to grow to hydroxyapatite-forming osteoblasts *ex vivo*. Nevertheless a thorough, continuous and intensive elaboration of the model in this line, to tailor a functionally polymer with self-regulatory activity on the bone cells, appears to be very encouraging. Animal trials are in progress.
